# Timing of vegetation sampling does not influence associations between visual obstruction and turkey nest survival in a montane forest

**DOI:** 10.1002/ece3.5681

**Published:** 2019-09-27

**Authors:** Michael J. Yarnall, Andrea R. Litt, Chadwick P. Lehman

**Affiliations:** ^1^ Department of Ecology Montana State University Bozeman MT USA; ^2^ South Dakota Game, Fish, and Parks Custer SD USA

**Keywords:** concealment, *Meleagris gallopavo*, nest survival, plant phenology, sampling bias, wild turkey

## Abstract

Evaluating relationships between ecological processes that occur concurrently is complicated by the potential for such processes to covary. Ground‐nesting birds rely on habitat characteristics that provide visual and olfactory concealment from predators; this protection often is provided by vegetation at the nest site. Recently, researchers have raised concern that measuring vegetation characteristics at nest fate (success or failure) introduces a bias, as vegetation at successful nests is measured later in the growing season (and has more time to grow) compared with failed nests. In some systems, this bias can lead to an erroneous conclusion that plant height is positively associated with nest survival. However, if the features that provide concealment are invariant during the incubation period, no bias should be expected, and the timing of measurement is less influential. We used data collected from 98 nests to evaluate whether there is evidence that such a bias exists in a study of wild turkey (*Meleagris gallopavo*) nesting in a montane forest ecosystem. We modeled nest survival as a function of visual obstruction and other covariates of interest. At unsuccessful nests, we collected visual obstruction readings at both the date of nest failure and the projected hatch date and compared survival estimates generated using both sets of vegetation data. In contrast to studies in grassland and shrubland systems, we found little evidence that the timing of vegetation sampling influenced conclusions regarding the association between visual obstruction and nest survival; model selection and estimates of nest survival were similar regardless of when vegetation data were collected. The dominant hiding cover at most of our nests was provided by evergreen shrubs; retention of leaves and slow growth of these plants likely prevent appreciable changes in visual obstruction during the incubation period. When considered in aggregate with a growing body of literature, our results suggest that the influence of timing of vegetation sampling depends on the study system. When designing future studies, investigators should carefully consider the type of structures that provide nest concealment and whether plant phenology is confounded with nest survival.

## Graphical abstract

We evaluated whether there is evidence that timing of vegetation sampling (nest fate vs. expected hatch date) introduces a temporal bias in a study of wild turkey (*Meleagris gallopavo*) nesting in a montane forest ecosystem. In contrast to studies in grassland and shrubland systems, we found little evidence that the timing of vegetation sampling influenced conclusions regarding the association between visual obstruction and nest survival; model selection and estimates of nest survival were similar regardless of when habitat data were collected.

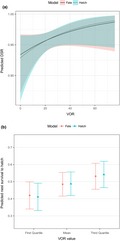

## 1 INTRODUCTION

Nest success is an important driver of population growth in avian species (Hoekman, Mills, Howerter, Devries, & Ball, 2002; Wisdom & Mills, 1997). Birds are expected to select nesting locations with features that maximize fitness (Hilden, 1965; Martin, 1993), but characterizing nest sites at the time of selection is challenging because nests may be difficult to locate at this early stage and because investigator disturbance may influence nest fate. Because predation is the most common cause of nest failure in many bird species (Lehman, Rumble, Flake, & Thompson, 2008; Martin, 1993; Ricklefs, 1969; Webb et al., 2012), habitat characteristics that provide visual and olfactory concealment from predators are commonly of interest in nest survival studies. However, support for the nest concealment hypothesis (Martin & Roper, 1988) in the literature is far from ubiquitous (Borgmann & Conway, 2015), likely because the factors that may influence the selection of nesting habitat and/or nest survival are both complex and numerous. Mismatches between habitat preferences and nesting outcomes continue to vex researchers, and numerous explanations have been proposed, including human disturbances (e.g., ecological traps, Chalfoun & Schmidt, 2012), ecological–evolutionary mechanisms (e.g., habitat preferences shaped by other fitness components or adaptive peaks, Borgmann & Conway, 2015; Chalfoun & Schmidt, 2012; Latif, Heath, & Rotenberry, 2012), and methods used to quantify habitat characteristics (e.g., variation in methods used to quantify concealment, Borgmann & Conway, 2015; Chalfoun & Schmidt, 2012).

Evaluating relationships between ecological processes that occur concurrently is complicated by the potential for such processes to covary. Vegetation often provides concealment at nests and nesting usually occurs during the spring growing season, so the influence of vegetation density and height on nest survival could be confounded with plant growth during the nesting period. Specifically, measuring vegetation following the termination of incubation (either nest failure or hatch, a method commonly used in nesting studies, Gibson, Blomberg, & Sedinger, 2016) may result in increased levels of concealment at successful nests simply because plants at successful nests will have, on average, more time to develop and produce cover than plants at failed nests. This sampling issue may overestimate the influence of vegetation on nest survival because of a relationship which is correlative, but not causative: Successful nests may be more concealed because they were sampled later, not because greater concealment lead them to be successful (Borgmann & Conway, 2015; Burhans & Thompson, 1998; Gibson et al., 2016; McConnell, Monroe, Burger, & Martin, 2017; Ringelman & Skaggs, 2019; Smith et al., 2018; Vega Rivera, Montaño, Rappole, & Cerda, 2009).

Although concern regarding bias introduced by the timing of vegetation sampling is long‐standing (Burhans & Thompson, 1998), sampling at nest fate has remained the norm in many investigations of nest survival (Gibson et al., 2016). Recently, Gibson et al. (2016) and McConnell et al. (2017) simulated data to demonstrate the potential to erroneously detect a positive association between vegetation height and nest survival even when no association existed or when the true association was negative. Additionally, these researchers found that the biased method was most commonly favored during model selection (Gibson et al., 2016; McConnell et al., 2017). Suggestions to avoid this confounding issue include date‐corrected estimates of vegetation measurements (Gibson et al., 2016) and measuring vegetation at the expected hatch date for failed nests (McConnell et al., 2017). Smith et al. (2018) used date‐corrected vegetation measurements to reanalyze data from multiple studies across the range of greater sage‐grouse (*Centrocercus urophasianus*) and, after correcting for plant phenology, found little evidence for a meaningful effect of grass height on the survival of sage‐grouse nests. A true‐positive effect of concealment was found for duck nests, but the effect size was over‐estimated when data were collected at nest fate (Ringelman & Skaggs, 2019). However, Ringelman and Skaggs (2019) also note that most studies of nest survival for ducks collect data on vegetation density at the time the nest is found, which is likely less biased. Additionally, vegetation density increased during the nesting period at successful nests, but decreased at failed nests, suggesting that in this system, measuring vegetation at the expected hatch date may introduce another source of bias (Ringelman & Skaggs, 2019).

**Figure 1:**
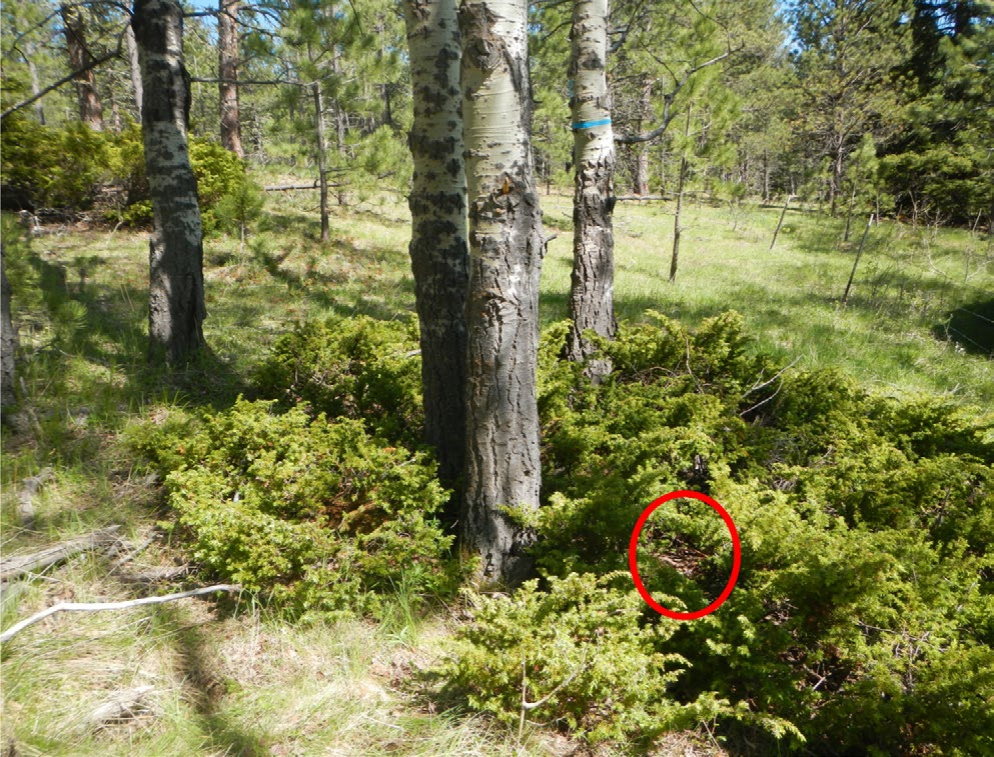
A typical turkey nest located in an evergreen shrub (common juniper, the most commonly used nesting cover), northern Black Hills, South Dakota. Red outline indicates the location of the nest bowl

In light of recent findings, more research is needed, particularly in vegetation communities other than grass and shrublands, as the influence of the timing of vegetation sampling may vary depending on the study system. Specifically, if vegetation and other features which provide concealment at the nest site do not change appreciably during the incubation period, collecting data solely at the termination of incubation should not influence the potential association between concealment and nest survival. We sought to build on the existing body of research using data from a montane forest ecosystem with wild turkeys (*Meleagris gallopavo*) and determine whether sampling visual obstruction at differing times (i.e., immediately following nest failure vs. projected hatch date) would introduce a bias as in other systems. Our objective was to compare estimates of nest survival and model selection results derived from sampling nest sites at two different times: (a) at date of nest failure and (b) at date of nest hatch for successful nests and at the projected hatch date for failed nests. Because nests in our study area frequently were concealed by slow‐growing evergreen vegetation (Figure 1) or nonvegetative features, we expected that the timing of sampling should have little to no impact on our inferences regarding the relationship between concealment and nest survival.

## 2 METHODS

### 2.1 Nesting data

We used data collected from wild turkey nests monitored during 2016 and 2017 in the northern Black Hills of South Dakota. We used radio telemetry to determine the onset of incubation behavior, after which we located nesting hens via homing and visual observation (White & Garrott, 1990) and recorded the approximate location using a handheld GPS. We marked at least three points surrounding the nest at approximately 30 m using survey tape and used a compass to record a bearing in the direction of the nest bowl. This facilitated location of the nest bowl following nest fate while also minimizing investigator disturbance. After nest marking, we checked each nest 1–2 times daily via radio telemetry to monitor success or failure (see Yarnall, 2019 for full details). All procedures were reviewed and approved by the Institutional Animal Care and Use Committee at Montana State University (protocol 2015‐25).

Because we were interested in how the timing of sampling might influence potential associations between visual obstruction and nest survival, we characterized nest sites within 1–2 days of nest fate for successful nests. Nests that failed were sampled twice: once within 1–2 days of nest fate and again within 1–2 days of the nest's expected hatch date. Due to logistical constraints, nests that failed within 4 days of their expected hatch date were sampled only once.

We marked four transects with survey tape along each cardinal direction centered at the nest bowl. To ensure measurements at failed nests were consistent between the date of nest failure and projected hatch dates, we left flagging in place between sampling periods. We measured visual obstruction readings (VOR) by placing a pole marked with 1.27‐cm increments in the nest bowl and recording the lowest visible increment when viewed from a height of 1 m and a distance of 4 m in each cardinal direction (Benkobi, Uresk, Schenbeck, & King, 2000; Robel, Briggs, Dayton, & Hulbert, 1970). Additionally, we collected VOR at a point 1 m from the nest bowl in each cardinal direction. For these points, we only recorded VOR from the three cardinal directions not across the nest bowl to avoid duplication (e.g., the 1 m north peripheral measurement was read from the E, N, and W). We averaged all VOR data collected at each nest (16 readings per nest) to create a single measure that described concealment at each nest. Because we thought that the influence of timing of sampling could vary depending on the primary vegetation type at the nest bowl, we used a Daubenmire frame (Daubenmire, 1959) to record understory canopy cover. We recorded coverage of grass, forbs, and shrubs at the nest bowl. We further classified the dominant shrub as deciduous or evergreen because the visual obstruction provided by a deciduous shrub might change if leaf budding occurred during incubation.

### 2.2 Nest survival analysis and model selection

We estimated daily survival rates (DSR) of nests using the nest survival model (Dinsmore, White, & Knopf, 2002; Rotella, Dinsmore, & Shaffer, 2004). We conducted analyses using Program MARK (White & Burnham, 1999) via RMark (Laake, 2013) in Program R (R Core Development Team, 2018). In a previous analysis of data from this system, daily precipitation, VOR, and year were included in the top model used to estimate DSR (Yarnall, 2019). We compared two models based on the most supported model from our previous analysis to evaluate whether potential changes in nest cover due to plant phenology would influence conclusions about the association between VOR and nest survival: One included VOR data collected immediately following the date of nest fate regardless of success/failure (hereafter the Full Fate model), and one that included VOR measurements collected following hatch at successful nests and at the projected hatch date for nests that failed (hereafter the Full Hatch model). Both these models also included daily precipitation and year. For comparison, we considered two models that allowed DSR to vary according to VOR alone: One included VOR data collected at nest fate (hereafter the VOR Fate model), and one included VOR data collected at the hatch date or following the projected hatch date (hereafter the VOR Hatch model). Additionally, we also included a null model (constant DSR).

Using an information theoretic approach (Burnham & Anderson, 2002), we compared the relative support for each of our five candidate models. To determine whether our inferences related to visual obstruction differed depending on the timing of sampling, we compared the magnitude of the effect size for visual obstruction and plotted predicted DSR across a range of VOR values from the Full Fate and Full Hatch models. Additionally, we used both the Full Fate and Full Hatch models to predict survival through a 26‐day incubation period to test whether sampling vegetation at fate (both hatch and failure) or hatch dates (both projected and actual) would result in differences in estimates of survival that were biologically meaningful. For survival to hatch estimates, we assumed incubation began on the median date of nest initiation, used daily precipitation amounts observed in 2017, and compared estimates for nests that had first quartile, mean, and third quartile values of VOR. We used the delta method to estimate standard errors of these estimates (Powell, 2007; Seber, 1982).

## 3 RESULTS

### 3.1 Nest monitoring and vegetation sampling

**Figure 2:**
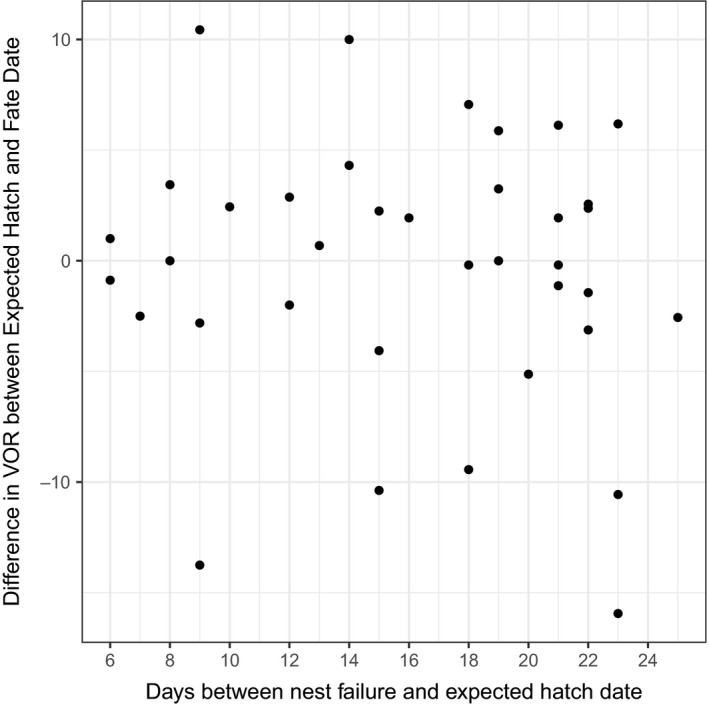
Change in mean visual obstruction reading (VOR, measured in 1.27‐cm increments) between projected hatch date and nest failure with respect to the number of days between vegetation sampling for unsuccessful turkey nests (*n* = 37) in the northern Black Hills, South Dakota, 2016–2017. Points above zero indicate a larger mean VOR when the nest was sampled at projected hatch date, relative to failure date; points below zero indicate mean VOR was larger when sampled at the failure date

We marked and monitored a total of 104 nests in 2016 and 2017, but omitted six nests from our survival analysis because we were unable to locate the nest bowl or collect data on nest site characteristics within 2 days of nest fate or projected hatch date. Of the 98 nests utilized in this analysis, 51 hatched and 47 failed. Of the 47 failed nests, 10 were sampled only once because they failed within 4 days of the projected hatch date. For the 37 nests that were sampled twice, the time between nest failure and projected hatch date was variable and ranged from 6 to 25 days (mean = 14.9 days, Figure 2).

Including both successful (sampled once) and failed nests (sampled twice), nest site characteristics were measured 135 times in surveys at 98 turkey nests, and grass or forb cover exceeded shrub cover in only 19 surveys (14%). In 15 of those surveys (11%), nonvegetative features (rock, slash, deadfall, tree trunks, etc.) provided the primary hiding cover. Among surveys where shrub cover met or exceeded grass or forb cover (116 surveys), the most common dominant shrub was evergreen; an evergreen species was the dominant shrub in 81 surveys (70%), the dominant shrub was deciduous at 33 nests (29%), and two nests (2%) were concealed by nonvegetative features and lacked a shrub of either type at the nest bowl. We found a similar pattern when we considered the 47 failed nests separately (84 surveys); grass or forb cover exceeded shrub cover in only 11 surveys (13%). Further, evergreen shrubs remained the most common type at failed nests (72%, 53 surveys); deciduous shrubs were dominant in 19 (26%) surveys, and one failed nest (1%) was constructed in a pine slash pile and did not have a shrub of either type.

**Figure 3:**
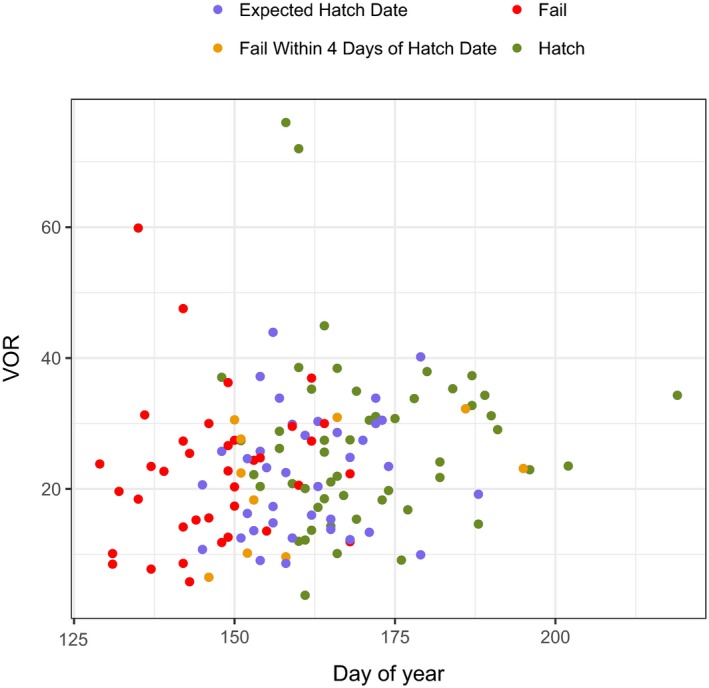
Mean visual obstruction reading (VOR, measured in 1.27‐cm increments) at wild turkey nests in the northern Black Hills, South Dakota, 2016–2017. Unsuccessful nests (red) were sampled following nest fate and at the projected hatch date (purple) unless the nest failed within 4 days of the expected hatch date (orange). Successful nests were sampled following hatch (green). We did not detect a relationship between VOR and day of year (adjusted *R* ^2^ = 0.02, $\hat{\beta }$_DayofYear_ = 0.11, *SE* = 0.06)

Although VOR at some nests did vary between sampling visits, we did not find systematic differences in VOR with respect to the timing of sampling (Figure 2). Mean VOR at nest failure was nearly identical to mean VOR at projected hatch date (*µ*_failure_ = 22.5, *SE* = 11.1; *µ*_projected hatch_ = 22.2, *SE* = 9.2; VOR measured in 1.27‐cm increments). Further, no trend in VOR was apparent with respect to date (Figure 3). This lack of pattern suggests that most differences between VOR at fate versus expected hatch date were due to sampling variation rather than major changes in vegetation providing concealment during incubation. VOR measured at the projected hatch date of failed nests was highly correlated with VOR measured at the date of nest failure (Pearson's correlation coefficient = 0.84). Mean VOR at successful nests (*µ*_successful_ = 26.9, *SE* = 11.5; VOR measured in 1.27‐cm increments) was slightly larger than VOR measured during either sampling event at failed nests.

### 3.2 Nest survival

We did not find evidence that the timing of vegetation sampling influenced our inferences regarding associations between visual obstruction and nest survival, given that there was similar support for both the Full Hatch and Full Fate models (Table 1). In both the Full models, estimated coefficients for VOR were similar regardless of sampling period: $\hat{\beta }$_Hatch VOR_ = 0.026 (*SE* = 0.016) and $\hat{\beta }$_Fate VOR_ = 0.022 (*SE* = 0.015). We found some evidence that larger VORs were associated with higher DSR. Adding VOR to the null model (the VOR Hatch and VOR Fate models) did improve model fit enough to overcome the penalty for the additional parameter. However, the coefficient was estimated too imprecisely to make an unequivocal statement regarding the magnitude of association between VOR and DSR (Table 1).

**Table 1:** Model selection results for nest survival models evaluating the association between visual obstruction and daily survival of turkey nests in the northern Black Hills, South Dakota, 2016–2017

Model	AIC_c_	ΔAIC_c_	AIC_c_ weight	No. parameters
Full hatch (Precipitation + VOR_Hatch_ + Year)	432.40	0.00	0.57	4
Full fate (Precipitation + VOR_Fate_ + Year)	432.97	0.57	0.42	4
VOR hatch (VOR_Hatch_)	439.52	7.13	0.02	2
VOR fate (VOR_Fate_)	440.22	7.82	0.01	2
Null	440.96	8.57	0.01	1

Note

Hatch models indicate that VOR data were collected following hatch or the projected hatch date. Fate models indicate that VOR data were collected following nest fate regardless of success or failure.

**Figure 4:**
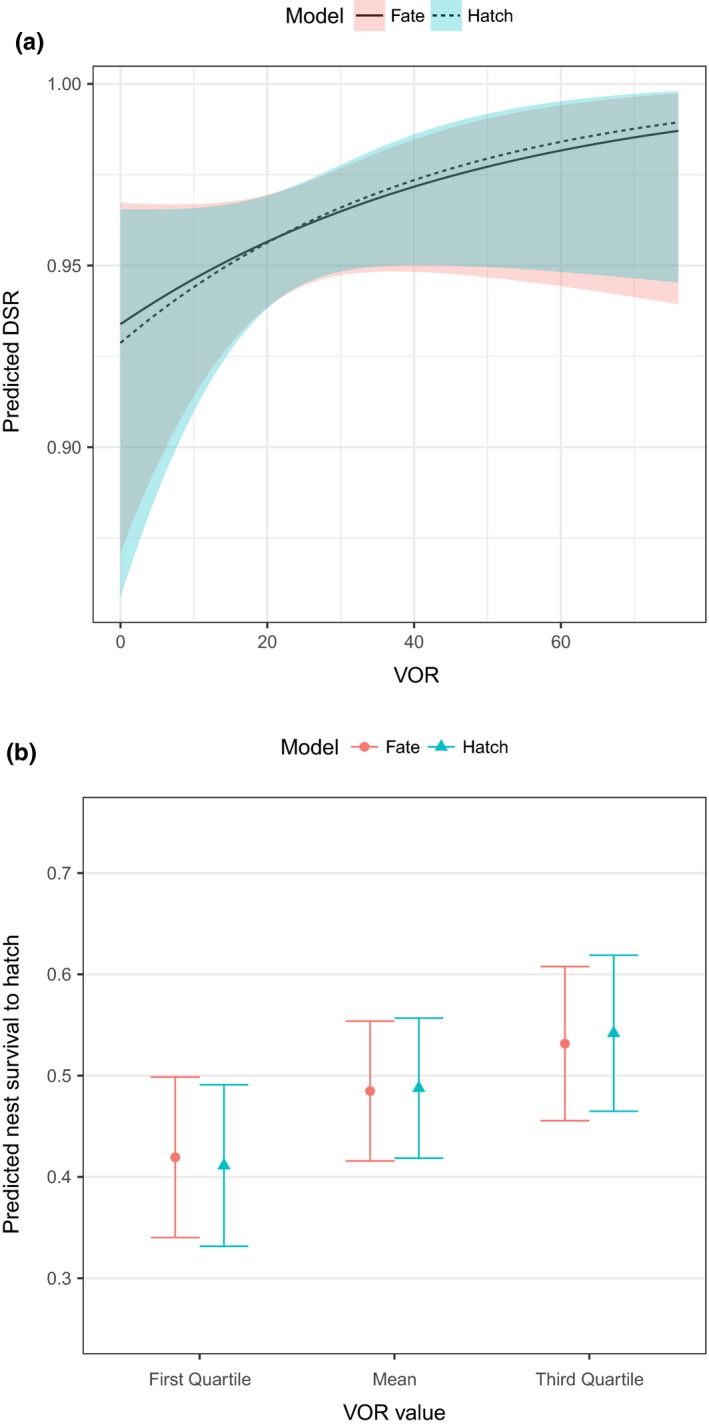
Predicted (a) daily survival rate (DSR; and 95% confidence intervals) across a range of visual obstruction values (VOR, measured in 1.27‐cm increments) and (b) survival (and standard errors) through incubation (26 days) comparing timing of nest measurement from the Hatch and Fate models for turkey nests (*n* = 98), 2016–2017, northern Black Hills, South Dakota

We plotted predicted DSR from both the Hatch and Fate sampling models across a range of VOR values with daily precipitation held at the mean of the nonzero precipitation values we observed (Figure 4a). Year was a simple additive effect, so we plotted DSR estimates for 2017. Estimated DSR values were similar, with near complete overlap in their 95% confidence intervals (Figure 4a). Although estimated nest survival to hatch varied based on visual obstruction, estimates were similar between the Hatch and Fate models (Figure 4b).

## 4 DISCUSSION

In contrast to other recent studies (Gibson et al., 2016; McConnell et al., 2017; Ringelman & Skaggs, 2019; Smith et al., 2018), we did not find evidence that timing of sampling (projected hatch date vs. failure date) influenced our estimates or inferences regarding the positive association between VOR and nest survival. Most nests were constructed in evergreen shrubs (primarily common juniper, *Juniperus communis*); retention of leaves and slow growth of these plants likely prevent appreciable changes in visual obstruction during the incubation period. The nonvegetative features (e.g., rocks, logs, pine slash) that provided the primary concealment at most nests not constructed within a shrub also were unlikely to change meaningfully during the incubation period. Similar to our findings, Hausleitner, Reese, and Apa (2005) found that only grass height and density varied when sage‐grouse nests were sampled at nest initiation and at hatch; other measures of vegetation concealment (e.g., VOR, nest shrub height) were consistent regardless of timing. Although grasses may contribute to VOR, changes in grass height or density are unlikely to result in meaningful changes to VOR, if concealment is primarily provided by other features. Evergreen shrubs in our study area frequently were sufficiently dense to exclude grass and forbs, so slow‐growing shrubs provided all or nearly all concealment of the nest, and faster growing plants that might change during nesting were of limited importance (Figure 1).

When considered in aggregate with a growing body of literature, our results suggest that the influence of timing of vegetation sampling, as well as the underlying relationship between concealment and nest survival, depends on the study system. Additionally, researchers' ability to detect the relationship varies among species. As noted by Ringelman and Skaggs (2019), many studies of duck nest survival measure vegetation when the nest is found because nests are located by flushing the female. This method is appropriate for some taxa (e.g., some waterfowl and songbirds), but not for species where investigator disturbance may induce nest abandonment. If researchers are unable to measure vegetation when the nest is found (preferably at nest initiation), there are multiple options to address the confounding influence of vegetation phenology: date‐corrected vegetation measurements (Gibson et al., 2016; Smith et al., 2018), measuring failed nests at the expected hatch date (McConnell et al., 2017), and restricting analysis to nests which were fated during the same time period (binning nests by fate date, Ringelman & Skaggs, 2019). Although Ringelman and Skaggs' (2019) date‐binning method enabled them to detect a difference in how vegetation density changed at successful versus failed nests, this method is impractical for studies that use radio telemetry to locate nests and are therefore unable to obtain the large sample sizes needed for this approach.

Because sampling nests at the initiation of incubation would influence nest fate for wild turkeys, we elected to control for the potential confounding effect of plant phenology by sampling failed nests at their projected hatch date. This sampling scheme does require an additional visit to the nest site, which is impractical for some studies (Ringelman & Skaggs, 2019). However, because our study was part of a larger effort to quantify turkey vital rates (Yarnall, 2019) and technicians were frequently near nesting sites already, we found that sampling at the projected hatch date was actually easier to incorporate into our field schedule than fate date sampling. Nests frequently fail unexpectedly, so sampling within 2 days of the nest's fate date often places added strain on field efforts. However, because the expected hatch date was known 6–25 days in advance, we were better able to plan for vegetation sampling. Additionally, the available window is longer for sampling at expected hatch date than fate date. That is, if the goal is to collect vegetation data within 2 days of the fate/expected hatch date, the sampling window for projected hatch date is 5 days (2 days prior, the expected hatch date, and 2 days after), whereas the sampling window for the fate date is only 3 days (the day the nest is fated, and 2 days after). This prescheduling and longer sampling period enabled greater flexibility to account for weather conditions, logistics, and other data collection needs. We suggest that this approach could ease logistical challenges for future studies, particularly those relying on radio‐marked females, with the added benefit of addressing the potential confounding effect of plant phenology for rigorous ecological inference.

Although we did not find evidence that visual obstruction differed based on timing of measurement in this system, this is unlikely to be the case in all vegetation communities for a species as wide ranging as wild turkeys. If hens rely more on herbaceous cover (e.g., hayfields, Shields & Flake, 2006) or use nest sites with a greater diversity of deciduous shrubs (e.g., Fuller, Spohr, Harrison, & Servello, 2013) that can rapidly grow leaves during nesting, measurements of VOR are more likely to change between nest failure and the projected hatch date. Unfortunately, nesting studies do not always provide clear descriptions of the methodology used to quantify concealment (Borgmann & Conway, 2015); we agree with Borgmann and Conway (2015) that investigators should provide more clarity when describing sampling methods. Given the crucial role of nest survival in the maintenance of bird populations (Hoekman et al., 2002; Wisdom & Mills, 1997), researchers will and should continue working to understand the underlying relationship between nesting habitat and nest survival. When designing future studies, investigators should carefully consider the types of structures that provide nest concealment, the species of interest, how plant phenology might be confounded with nest survival, and how to best address potential confounding. Further, environmental covariates that vary through time may introduce bias to other ecological responses of interest due to the timing of sampling (see Gibson et al., 2016 for an in‐depth discussion of the issue beyond nest survival). Ensuring covariates accurately represent ecological phenomena will help provide rigorous inferences to develop management plans and conserve species.

## CONFLICT OF INTEREST

None declared.

## AUTHOR CONTRIBUTIONS

MJY collected, organized, and managed data, conceptualized the study, conducted the analysis, produced figures, and wrote the manuscript. ARL guided study design, helped secure funding, assisted with data analysis, and provided extensive review of the manuscript and early drafts. CPL secured funding, assisted with data collection, and reviewed the manuscript. All authors revised and approved the final manuscript draft.

## Data Availability

Nesting data presented in this paper are available at Dryad: https://doi.org/10.5061/dryad.g7r412v.

## References

[ece35681-bib-0001] Benkobi, L. , Uresk, D. W. , Schenbeck, G. , & King, R. M. (2000). Protocol for monitoring standing crop in grasslands using visual obstruction. Journal of Range Management, 53, 627–633. 10.2307/4003158

[ece35681-bib-0002] Borgmann, K. L. , & Conway, C. J. (2015). The nest‐concealment hypothesis: New insights from a comparative analysis. The Wilson Journal of Ornithology, 127(4), 646–660.

[ece35681-bib-0003] Burhans, D. E. , & Thompson, F. R. (1998). Effects of Time and Nest‐Site Characteristics on Concealment of Songbird Nests. The Condor, 100(4), 663–672.

[ece35681-bib-0004] Burnham, D. A. , & Anderson, K. P. (2002). Model selection and multi‐model inference: A practical information‐theoretic approach (2nd ed., Vol. 172). New York, NY: Springer.

[ece35681-bib-0005] Chalfoun, A. D. , & Schmidt, K. A. (2012). Adaptive breeding‐habitat selection: Is it for the birds? The Auk, 129, 589–599. 10.1525/auk.2012.129.4.589

[ece35681-bib-0006] Daubenmire, R. (1959). A canopy‐coverage method of vegetational analysis. Northwest Science, 33, 43–46.

[ece35681-bib-0007] Dinsmore, S. J. , White, G. C. , & Knopf, F. L. (2002). Advanced techniques for modeling avian nest survival. Ecology, 83, 3476–3488. 10.1890/0012-9658(2002)083[3476:ATFMAN]2.0.CO;2

[ece35681-bib-0008] Fuller, A. K. , Spohr, S. M. , Harrison, D. J. , & Servello, F. A. (2013). Nest survival of wild turkeys *Meleagris gallopavo silvestris* in a mixed‐use landscape: Influences at nest‐site and patch scales. Wildlife Biology, 19, 138–146.

[ece35681-bib-0009] Gibson, D. , Blomberg, E. J. , & Sedinger, J. S. (2016). Evaluating vegetation effects on animal demographics: The role of plant phenology and sampling bias. Ecology and Evolution, 6, 3621–3631. 10.1002/ece3.2148 27148444 PMC4848082

[ece35681-bib-0010] Hausleitner, D. , Reese, K. P. , & Apa, A. D. (2005). Timing of vegetation sampling at greater sage‐grouse nests. Rangeland Ecology & Management, 58, 553–556. 10.2111/04-170R2.1

[ece35681-bib-0011] Hilden, O. (1965). Habitat selection in birds: A review. Finnish Zoological and Botanical Publishing Board, 2, 53–75.

[ece35681-bib-0012] Hoekman, S. T. , Mills, L. S. , Howerter, D. W. , Devries, J. H. , & Ball, I. J. (2002). Sensitivity analyses of the life cycle of midcontinent mallards. The Journal of Wildlife Management, 66, 883–900. 10.2307/3803153

[ece35681-bib-0013] Laake, J. L. (2013). RMark: An R interface for analysis of capture‐recapture data with MARK. AFSC Processed Rep. 2013‐01. Seattle, WA: Alaska Fisheries Science Center, NOAA, National Marine Fisheries Service.

[ece35681-bib-0014] Latif, Q. S. , Heath, S. K. , & Rotenberry, J. T. (2012). How avian nest site selection responds to predation risk: Testing an ‘adaptive peak hypothesis’. Journal of Animal Ecology, 81, 127–138. 10.1111/j.1365-2656.2011.01895.x 21848943

[ece35681-bib-0015] Lehman, C. P. , Rumble, M. A. , Flake, L. D. , & Thompson, D. J. (2008). Merriam's turkey nest survival and factors affecting nest predation by mammals. Journal of Wildlife Management, 72, 1765–1774. 10.2193/2007-519

[ece35681-bib-0016] Martin, T. E. (1993). Nest predation and nest sites: New perspectives on old patterns. BioScience, 43, 523–532. 10.2307/1311947

[ece35681-bib-0017] Martin, T. E. , & Roper, J. J. (1988). Nest predation and nest‐site selection of a western population of the hermit thrush. The Condor, 90, 51–57. 10.2307/1368432

[ece35681-bib-0018] McConnell, M. D. , Monroe, A. P. , Burger, L. W. , & Martin, J. A. (2017). Timing of nest vegetation measurement may obscure adaptive significance of nest‐site characteristics: A simulation study. Ecology and Evolution, 7, 1259–1270. 10.1002/ece3.2767 28303194 PMC5306001

[ece35681-bib-0019] Powell, L. (2007). Approximating variance of demographic parameters using the Delta Method: A reference for avian biologists. The Condor, 109, 949–954.

[ece35681-bib-0020] R Core Development Team (2018). R: A language and environment for statistical computing. Vienna, Austria: R Foundation for Statistical Computing.

[ece35681-bib-0021] Ricklefs, R. E. (1969). An analysis of nesting mortality in birds. Smithsonian Contributions to Zoology, (9), 1–48. 10.5479/si.00810282.9

[ece35681-bib-0022] Ringelman, K. M. , & Skaggs, C. G. (2019). Vegetation phenology and nest survival: Diagnosing heterogeneous effects through time. Ecology and Evolution, 9, 2121–2130. 10.1002/ece3.4906 30847097 PMC6392373

[ece35681-bib-0023] Robel, R. J. , Briggs, J. N. , Dayton, A. D. , & Hulbert, L. C. (1970). Relationships between visual obstruction measurements and weight of grassland vegetation. Journal of Range Management, 23, 295–297. 10.2307/3896225

[ece35681-bib-0024] Rotella, J. J. , Dinsmore, S. J. , & Shaffer, T. L. (2004). Modeling nest‐survival data: A comparison of recently developed methods that can be implemented in MARK and SAS. Animal Biodiversity and Conservation, 27, 187–205.

[ece35681-bib-0025] Seber, G. A. F. (1982). The estimation of animal abundance and related parameters (2nd ed.). London, UK: Chapman and Macmillan, New York.

[ece35681-bib-0026] Shields, R. D. , & Flake, L. D. (2006). Survival and reproduction of translocated eastern wild turkeys in a sparsely wooded landscape in north eastern South Dakota. Western North American Naturalist, 66, 298–309. 10.3398/1527-0904(2006)66[298:SAROTE]2.0.CO;2

[ece35681-bib-0027] Smith, J. T. , Tack, J. D. , Doherty, K. E. , Allred, B. W. , Maestas, J. D. , Berkeley, L. I. , … Naugle, D. E. (2018). Phenology largely explains taller grass at successful nests in greater sage‐grouse. Ecology and Evolution, 8, 356–364. 10.1002/ece3.3679 29321877 PMC5756841

[ece35681-bib-0028] Vega Rivera, J. H. , Montaño, I. M. , Rappole, J. , & Cerda, F. C. (2009). Testing the importance of nest concealment: Does timing matter? Journal of Field Ornithology, 80, 303–307. 10.1111/j.1557-9263.2009.00234.x

[ece35681-bib-0029] Webb, S. L. , Olson, C. V. , Dzialak, M. R. , Harju, S. M. , Winstead, J. B. , & Lockman, D. (2012). Landscape features and weather influence nest survival of a ground‐nesting bird of conservation concern, the greater sage‐grouse, in human‐altered environments. Ecological Processes, 1, 1–15. 10.1186/2192-1709-1-4

[ece35681-bib-0030] White, G. C. , & Burnham, K. P. (1999). Program MARK: Survival estimation from populations of marked animals. Bird Study, 46(Supplement), 120–138. 10.1080/00063659909477239

[ece35681-bib-0031] White, G. C. , & Garrott, R. A. (1990). Analysis of wildlife radio‐tracking data. San Diego, CA: Academic Press Inc.

[ece35681-bib-0032] Wisdom, M. J. , & Mills, L. S. (1997). Sensitivity analysis to guide population recovery: Prairie‐chickens as an example. The Journal of Wildlife Management, 61, 302–312. 10.2307/3802585

[ece35681-bib-0033] Yarnall, M. J. (2019). Impacts of weather, habitat, and reproduction on the survival and productivity of wild turkeys in the northern Black Hills, South Dakota. Thesis, Montana State University, Bozeman.

